# Epicardial Transplantation of Autologous Cardiac Micrografts During Coronary Artery Bypass Surgery

**DOI:** 10.3389/fcvm.2021.726889

**Published:** 2021-09-14

**Authors:** Annu Nummi, Severi Mulari, Juhani A. Stewart, Sari Kivistö, Kari Teittinen, Tuomo Nieminen, Milla Lampinen, Tommi Pätilä, Harri Sintonen, Tatu Juvonen, Markku Kupari, Raili Suojaranta, Esko Kankuri, Ari Harjula, Antti Vento, the AADC consortium

**Affiliations:** ^1^Heart and Lung Center, Helsinki University Hospital and University of Helsinki, Helsinki, Finland; ^2^Department of Radiology, Helsinki University Hospital (HUS) Medical Imaging Center and Helsinki University Hospital and University of Helsinki, Helsinki, Finland; ^3^Department of Internal Medicine, Päijät-Häme Central Hospital, Lahti, Finland; ^4^Department of Pharmacology, Faculty of Medicine, University of Helsinki, Helsinki, Finland; ^5^Pediatric Cardiac Surgery, Children's Hospital, Helsinki University Hospital and University of Helsinki, Helsinki, Finland; ^6^Department of Public Health, University of Helsinki, Helsinki, Finland; ^7^Department of Anesthesiology and Intensive Care, Helsinki University Hospital and University of Helsinki, Helsinki, Finland

**Keywords:** atrial appendage, autologous micrografts, cell therapy, coronary artery bypass surgery, epicardial cell delivery, ischemic heart failure

## Abstract

**Background:** Cardio-regenerative cell therapies offer additional biologic support to coronary artery bypass surgery (CABG) and are aimed at functionally repairing the myocardium that suffers from or is damaged by ischemia. This non-randomized open-label study assessed the safety and feasibility of epicardial transplantation of atrial appendage micrografts (AAMs) in patients undergoing CABG surgery.

**Methods:** Twelve consecutive patients destined for CABG surgery were included in the study. Six patients received AAMs during their operation and six patients were CABG-operated without AAMs transplantation. Data from 30 elective CABG patients was collected for a center- and time-matched control group. The AAMs were processed during the operation from a biopsy collected from the right atrial appendage. They were delivered epicardially onto the infarct scar site identified in preoperative late gadolinium enhancement cardiac magnetic resonance imaging (CMRI). The primary outcome measures at the 6-month follow-up were (i) patient safety in terms of hemodynamic and cardiac function over time and (ii) feasibility of therapy administration in a clinical setting. Secondary outcome measures were left ventricular wall thickness, change in myocardial scar tissue volume, changes in left ventricular ejection fraction, plasma concentrations of N-terminal pro-B-type natriuretic peptide levels, NYHA class, number of days in hospital and changes in the quality of life.

**Results:** Epicardial transplantation of AAMs was safe and feasible to be performed during CABG surgery. CMRI demonstrated an increase in viable cardiac tissue at the infarct site in patients receiving AAMs treatment.

**Conclusions and Relevance:** Transplantation of AAMs shows good clinical applicability as performed during cardiac surgery, shows initial therapeutic effect on the myocardium and has the potential to serve as a delivery platform for cardiac gene therapies.

**Trial Registration:**ClinicalTrials.gov, identifier: NCT02672163.

## Introduction

Coronary artery bypass graft (CABG) surgery reinstates myocardial blood flow downstream of an occluded coronary artery. Although CABG provides the patient with symptomatic benefit, it unfortunately does not restore the cells lost to infarction (1, 2). Additional therapies, including regenerative cell transplantation, have therefore been investigated for decades, but none have so far been adopted for clinical use (3–5). Furthermore, many regenerative therapies are associated with lengthy processing times and significant costs (6).

The current consensus for a CABG-supportive cardio-regenerative therapy is that the treatment should consist of a mixture of cardiac cell types and their extracellular matrix in a synergistic composition (7–9). To this end, we have demonstrated the preclinical efficacy and initial clinical feasibility (10) of autologous cardiac microtissue, atrial appendage micrografts (AAMs), and have shown that epicardial AAMs therapy activates cardiogenic and cardioprotective pathways (11). Based on these encouraging results, we initiated an open-label, non-randomized clinical study (12) to evaluate the safety and feasibility of AAMs transplantation in conjunction with CABG surgery. We utilized late gadolinium enhancement cardiac magnetic resonance imaging (CMRI) before and 6 months after treatment to gain detailed insight into the AAMs' efficacy to induce myocardial repair.

## Materials and Methods

### Ethics and Patient Selection

The study protocol was evaluated and approved by the Surgical Ethics Committee of the Hospital District of Helsinki and Uusimaa (number 180/13/03/02/13). The study is registered in the ClinicalTrials.gov database with the identification number NCT02672163. For the interventional open label non-randomized study, a total of 12 patients scheduled for elective CABG surgery from the Helsinki University Hospital (Helsinki, Finland) were recruited in chronological order. An additional 30 patients served as site- and time-matched reference controls. Similar to our earlier clinical cell therapy trial which assessed the effects of bone marrow mononuclear cell transplantation (13, 14), patients of either gender were evaluated for participation if they had ischemic heart failure and were scheduled for elective CABG. The criteria for inclusion and exclusion of the patients are presented in Table 1. Each patient was given both oral and written information about the trial, and the patient's signed informed consent was required for participation. After recruitment and drug optimization, patients waited 4–12 weeks for the elective operation. During this time, the ejection fraction (EF) changed in the AAMs group and control group.

**Table 1:** Criteria for patient selection—inclusion and exclusion criteria for patients enrolled in the study.

**Criteria for eligibility**
**Inclusion criteria**
1	Stable coronary artery disease filling the criteria for bypass surgery
2	Age between 18 and 75 years
3	Informed consent obtained
4	LVEF between ≤50 and ≥15%
5	NYHA Class II–IV heart failure symptoms
**Exclusion criteria**
1	Heart failure due to LV outflow tract obstruction
2	History of life-threatening ventricular arrhythmias or resuscitation, a condition possibly repeating, or an implantable cardioverter-defibrillator
3	Stroke or other disabling condition within 3 months before screening
4	Severe valvular disease or scheduled valvular surgery
5	Renal dysfunction (GFR <84 ml/min/1.73 m^2^)
6	Other disease limiting life expectancy
7	Contraindications for coronary angiogram or CMRI
8	Participation in some other clinical trial

*Abbreviations: CMR, cardiac magnetic resonance; LVEF, left ventricular ejection fraction; NYHA, New York Heart Association; LV, left ventriculum; GFR, glomerular filtration rate*.

The first six patients were recruited to the AAMs group and they received the AAMs transplant during CABG surgery. The next six patients formed the Control group I. Their surgeries were performed according to the normal hospital protocol and without AAMs transplantation. These two groups underwent the same follow-up protocol. To determine the safety of the procedure, the following 30 patients who met the study's inclusion and exclusion criteria, and were scheduled for elective CABG operation, formed Control group II. These patients were treated according to normal hospital protocol, without the AAMs transplant or any additional imaging, examination or blood tests as were required for the prior two groups.

**Figure 1:**
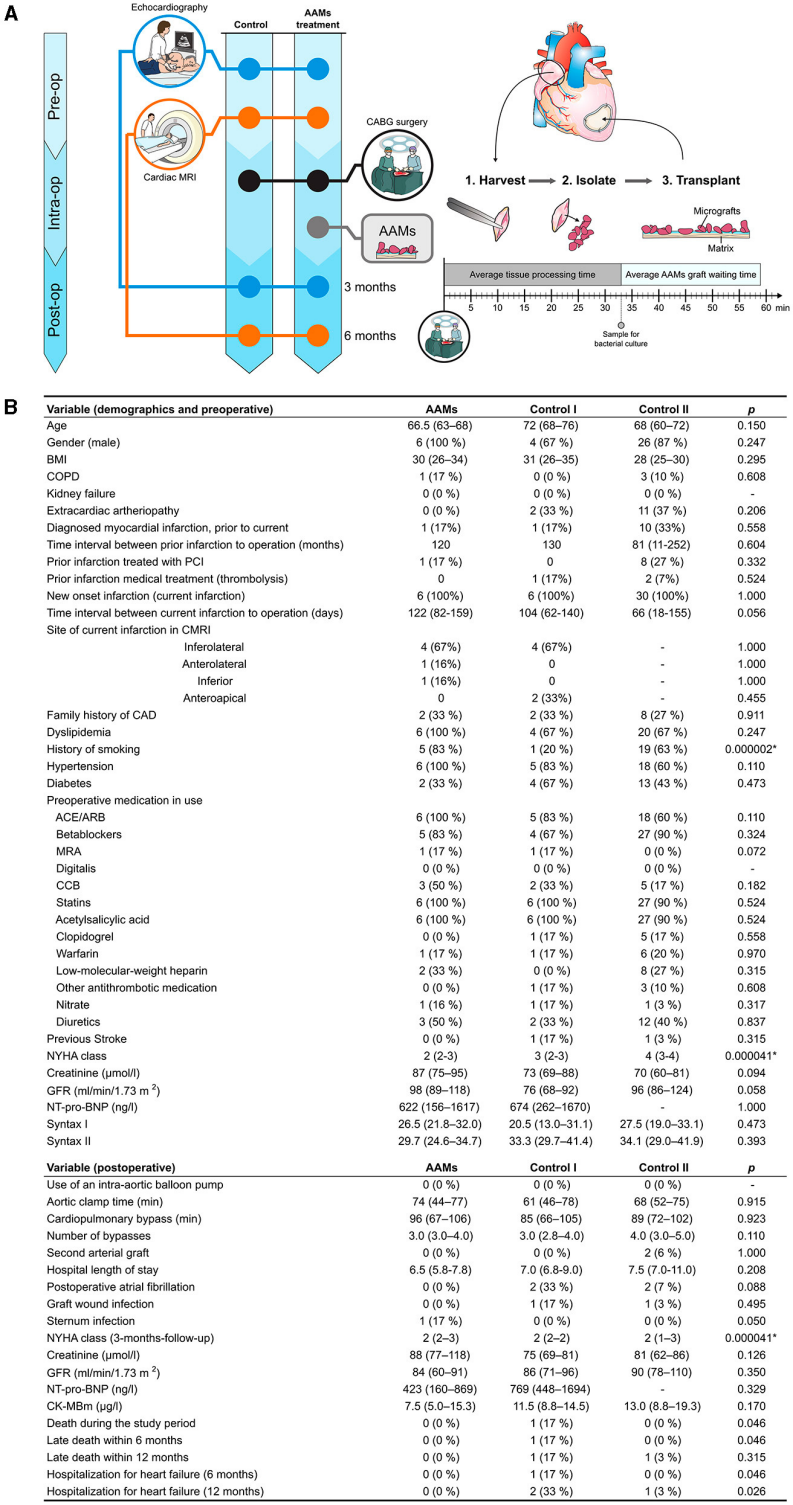
Study outline and demographics—study outline and demographics with pre-operative and post-operative data. **(A)** All patients underwent pre-operative (Pre-op) echocardiography and CMRI. The Study group received AAMs therapy during coronary artery bypass (CABG) surgery, while the Control group I (Control I) only underwent CABG surgery. AAMs were harvested from the right atrial appendage, mechanically isolated, and transplanted intraoperatively (Intra-op). Patients in both groups were examined 3-months post-operatively (Post-op) by echocardiography and 6 months after the operation using CMRI. **(B)** Demographics, pre- and post-operative data of the AAMs group (AAMs) and both control groups (Control I and Control II). ^1^ACE, angiotensin-converting enzyme inhibitor; ARB, angiotensin receptor blocker; BMI, body mass index; CAD, coronary artery disease; CCB, calcium channel blockers; CK-MB, creatine kinase myocardial band; CMRI, cardiac magnetic resonance imaging; COPD, chronic obstructive pulmonary disease; GFR, glomerular filtration rate; MRA, mineralocorticoid receptor antagonists; NT-Pro-BNP, N-terminal pro-B-type natriuretic peptide; NYHA, New York Heart Association; PCI, percutaneous coronary intervention. Results are presented as median (IQR), group comparisons were performed with the Kruskal-Wallis test. Mann Whitney *U*-test was used for the variables in only Study and Control group I results, or Chi Square for ordinal variables. Significant results after correction (Bonferroni) for multiple comparison are marked by an asterisk (*), with the results of *post-hoc* between-group analyses found in the text.

Echocardiography (echo), New York Heart Association (NYHA) class, basic laboratory tests, and blood N-terminal pro-B-type natriuretic peptide (NT-pro-BNP) concentrations were evaluated at baseline and 3-month follow-up for the patients in the AAMs group and Control group I. CMRI was performed and quality of life (QoL), measured using the health-related questionnaire instrument 15D (15), was evaluated twice per patient: preoperatively and at the 6-month follow-up. The study outline is shown in Figure 1A.

### Micrograft Isolation

During CABG surgery, AAMs were harvested from the right atrial appendage. A small piece of atrial appendage was removed at the site of insertion for a venous cannula for the heart-and-lung machine. The size and quality of the atrial appendage differ between patients according to age, gender, and comorbidities. To standardize the obtainable size of atrial appendage tissue, a minimum length of 5 × 10 mm and weight from 600 to 800 mg was required for each sample.

The harvested tissue was processed on-site in the operating room using a cell therapy tissue homogenizer (Rigenera-system, HBW s.r.l., Turin, Italy). Cell isolation was performed by a nurse who had received training in a cell-culture laboratory and in our previous studies by using a large-animal model (manuscript in preparation, Nummi et al.). Isolation was performed under strict adherence to sterility. We have previously described the preparation of the AAM transplant in detail (10, 12). As quantified earlier (10), the cell yield from this procedure is 9.76 × 10^6^ ± 0.53 × 10^6^ cells/g of tissue. Cell viability was 90.6% (10).

The isolated AAMs were applied in cardioplegia suspension to an extracellular matrix sheet (Cormatrix® ECMTM Technology, Cormatrix Cardiovascular Inc., Atlanta, GA, USA). Fibrin sealant (Tisseel^TM^, Baxter Healthcare Corp. Westlake Village, CA, USA), routinely used in surgery as tissue glue, was added to the cell suspension to secure the AAMs to the matrix.

### Therapy Administration

A standard CABG operation was performed under cardiopulmonary bypass and mild hypothermia, where patients were under cardiac arrest and receiving cardioplegia protection. After completion of the bypass anastomoses, the AAMs transplant was placed over the infarct scar area as determined preoperatively from each individual patient's pre-CABG CMRI images. The sheet with AAMs was placed on top of the damaged myocardium so that the micrografts were facing the epicardium. The matrix sheet attached well to the myocardial surface, and was further secured by three to four simple sutures with non-absorbable monofilament polypropylene string. The therapy application procedure was carefully photographed during each surgery, and the treatment administration site was meticulously detailed in patient documents for further CMRI and echo analyses.

### Tests for Transplant Sterility

To verify sterility, samples for microbial cultures were taken from each AAM-transplant.

### Clinical Cardiac Magnetic Resonance Imaging

CMRI was performed with a 1.5 T Avanto fit scanner and phase array cardiac coil (Siemens, Erlangen, Germany). Images were electrocardiography-gated and taken during breath-holding. left ventricle (LV) structure and function were imaged by a standardized CMRI protocol. TrueFISP cine series were obtained at the vertical and horizontal long axis for scouts to line up short-axis images. The stack of short-axis images was obtained from the mitral valve plane through the apex. To detect the myocardial scar, late gadolinium enhancement (LGE) was imaged with a 2D-segmented inversion recovery gradient echo sequence 12–20 min after Dotarem® injection (279.3 mg/ml; dose 0.2 mmol/kg). LGE images were obtained for the same views and slice/gap thickness as cine imaging.

All images were analyzed using a dedicated workstation (Medis® suite 3.2.28.0. Medis Medical Imaging Systems, Leiden, the Netherlands). LV function and volumetry were assessed from cine images with Qmass Analyse Program analysis software (Medis) and global longitudinal strain with Qstrain (Medis). Full width with half maximum (FWHM) technique and signal threshold vs. reference mean (STRM) threshold of 5 standard deviations (SD) above the mean signal intensity (SI) was used to assess myocardial and infarcted area mass. The thickness of infarction was measured from the thinnest point exactly at the same position from both images.

### End-Point Measures

The primary outcome measures were patient safety in terms of hemodynamic and cardiac function and feasibility of the therapy administration in a clinical setting. Hemodynamics were evaluated during each patient's operation and stay at the intensive care unit (ICU) given requirement for vasoactive medication and success of weaning from cardiopulmonary bypass and respirator. Post-operative hemodynamic criteria for assessing safety were cardiac index, hemoglobin, central venous oxygenation (SvO_2_), serum potassium level, blood glucose and lactate levels, and arterial pH. Cardiac function was evaluated during the operation by transesophageal echo and during the patient's stay at the ICU by transthoracic echo as well as constant telemetric monitoring of rhythm. Criteria for peri- and post-operative myocardial infarctions were: new regional wall motion abnormality confirmed by echo, ck-mb >50 μg/L, ischemic changes in ECG (LBBB or Q-waves), ventricular arrhythmia, or angiographically documented new graft or new native coronary artery occlusion. Feasibility was evaluated by the success in completing the delivery of the AAMs transplant to the myocardium, waiting times in minutes for the AAMs transplant and the success in closing the right atrial appendage by purse-string suture without additional sutures or patching.

The secondary outcome measures were change in LV infarct area thickness, movement and diastolic function assessed by CMRI, change in the amount of myocardial scar tissue as assessed by CMRI, local changes in systolic, and diastolic measures as estimated by echo, changes in LV ejection fraction (LVEF), NT-pro-BNP level, NYHA class, hospitalization or the days in hospital, and QoL.

### Statistics

Results are given as median with interquartile ranges (IQR), or *n* (%) of group for ordinal and nominal values. Comparisons between groups were performed with the Mann Whitney *U*-test, or between three groups with the Kruskal-Wallis test. Ordinal variables were tested with the Chi Square test. Multiple comparisons were corrected with the Bonferroni method, significant findings were further tested groupwise using the Mann Whitney *U*-test or Chi Square test, as applicable. Quality of life data are presented as mean (SD), and were analyzed with the independent samples *t*-test (two-sided). The level of significance (α) was 0.05. Analyses were performed with the IBM SPSS Statistics 25 program (IBM Corp., Armonk, NY).

## Results

Patient demographics are presented in Figure 1B. The median NYHA class differed between the AAMs group and Control group II [median NYHA 2 (range 2–3) in the AAMs group and median 4 (range 2–4) in the Control group II; *p* < 0.0001]. Control group I had NYHA median 3 (range 2–3). A history of smoking was more common in the AAMs group (*n* = 5, 83%) than in the control groups (Control I, *n* = 1, 20%; Control II, *n* = 19, 63%; *p* < 0.0001). Otherwise, there were no significant differences between the groups.

All atrial appendages were closed without difficulties with a single purse-string suture. The AAMs transplant was easily prepared in the operating room during CABG surgery. The median time for preparing the AAMs transplant was 33 min (range 22–43 min), and the median waiting time for the transplant was 26 min (range 7–54 min). In every operation, the AAMs transplant was ready to be placed before the anastomosis was done. In the AAMs group there were 4 inferolateral, 1 anterolateral, and 1 inferior and in Control I group 4 inferolateral and 2 anteroapical wall infarcts. All transplants were secured with three to four sutures without difficulties. Bacterial cultures taken from each AAMs transplant were negative for bacterial growth, demonstrating the sterility of the procedure.

Physiological data from the ICU at admission and 24 h after are shown in Table 2. Despite a significant difference in 24 h lactate concentrations between the three groups, all lactate values were within normal range (0.8–1.1 mmol/L). Echo measurements and ECG findings demonstrated no significant differences between the study groups (Table 3).

**Table 2:** Physiological data from ICU—physiological data from the first 24 h of the ICU, comparing the study group to the two control groups.

**Variable**	**Time**	**AAMs**	**Control I**	**Control II**	***p***
Heart Rate (min^−1^)	0 h	53 (42–69)	73 (61–82)	85 (78–89)	0.030
CVP (cmH_2_0)		8 (5–9)	8 (4–11)	10 (6–13)	0.293
Systolic arterial pressure (mmHg)		112 (106–136)	113 (96–129)	105 (93–113)	0.138
MAP (mmHg)		79 (71–87)	74 (65–83)	67 (63–77)	0.128
Diastolic arterial pressure (mmHg)		61 (55–66)	60 (47–62)	55 (48–59)	0.094
SpO_2_ (%)		100 (99–100)	100 (94–100)	99 (98–100)	0.368
aB-BE		0.1 (−1.0 to 0.8)	−2.2 (−3.0 to −0.6)	−0.7 (−2.1 to 0.8)	0.166
Lactate (mmol/l)		1.3 (1.2–1.8)	1.3 (0.9–1.6)	1.0 (0.8–1.4)	0.188
Hb (g/l)		136 (123–141)	128 (93–128)	115 (99–133)	0.162
Glucose (mmol/l)		7.1 (6.4–9.4)	9.0 (6.6–12)	7.3 (6.6–10.3)	0.681
Heart Rate (min^−1^)	24 h	82 (77–92)	85 (78–95)	82 (78–90)	0.793
CVP (cmH_2_0)		10 (8–13)	7 (5–10)	10 (6–14)	0.342
Systolic arterial pressure (mmHg)		143 (120–157)	123 (118–137)	114 (102–130)	0.009
MAP (mmHg)		87 (77–88)	75 (73–82)	69 (66–81)	0.043
Diastolic arterial pressure (mmHg)		61 (56–62)	55 (51–61)	52 (46–59)	0.112
SpO_2_ (%)		97 (96–99)	99 (96–99)	98 (96–99)	0.751
aB-BE		−1.0 (−1.7 to 0.6)	−1.6 (−2.3 to −0.25)	−1.5 (−3.2 to −0.2)	0.451
Lactate (mmol/l)		1.0 (0.8–1.1)	0.6 (0.6–1.0)	1.2 (1.0–1.7)	0.005
Hb (g/l)		99 (93–108)	111 (92–120)	107 (96–115)	0.590
Glucose (mmol/l)		8.2 (7.7–8.3)	8.7 (7.1–12.3)	8.1 (7.0–8.9)	0.651

*No significant differences were noted between the three groups. Results presented as median (IQR), group comparison performed with the Kruskal-Wallis test and Bonferroni correction (corrected significance level P = 0.001)*.

*Abbreviations: CVP, central venous pressure; MAP, mean arterial pressure; SpO_2_, peripheral capillary oxygen saturation; ab-Be, arterial blood base excess; Hb, hemoglobin*.

**Table 3:** Cardiac ultrasound and electrocardiogram data—cardiac ultrasound and electrocardiogram data from the preoperative baseline to the 3-month check-up visit, with comparisons of the study group vs. both control groups.

**Baseline**	**AAMs**	**Control I**	**Control II**	***p***
LV EF (%)	45 (35–53)	35 (35–45)	40 (20–60)	0.337
LVEDD (mm)	60 (51–67)	60 (45–61)	57 (42–70)	0.887
LA (mm)	44 (40–56)	45 (32–50)	41 (32–49)	0.527
E wave (m/s)	0.6 (0.5–0.9)	1.0 (0.8–1.2)	0.7 (0.4–1.1)	0.279
A wave (m/s)	0.6 (0.3–0.7)	0.5 (0.5)	0.6 (0.3–0.7)	0.721
ECG: Q Wave	1 (17 %)	3 (50 %)	15 (50 %)	0.264
ECG: LBBB	1 (17 %)	1 (17 %)	1 (3 %)	0.280
**3 month follow-up**	**AAMs**	**Control I**	**Control II**	***p***
LV EF (%)	46.5 (35–60)	40 (30–45)	50 (27–65)	0.312
LVEDD (mm)	56.5 (49–60)	55 (45–61)	56 (40–70)	0.980
LA (mm)	44.5 (38–50)	48 (30–49)	44 (35–53)	0.930
E (m/s)	0.5 (0.4–1.0)	0.7 (0.7–0.8)	0.7 (0.4–1.2)	0.557
A (m/s)	0.5 (0.5–0.9)	0.9 (0.8–0.9)	0.7 (0.2–1.3)	0.152
ECG: Q Wave	2 (33 %)	4 (67 %)	18 (60 %)	0.264
ECG: LBBB	1 (17 %)	1 (17 %)	2 (7 %)	0.536

*No significant differences were noted between groups. Results presented as median (range), group comparison performed with the Kruskal-Wallis test or Chi Square test (ordinal variables) with Bonferroni correction (corrected significance level p = 0.007)*.

*Abbreviations: ECG, electrocardiogram; EF, ejection fraction; LA, left atrium; LBBB, left bundle branch block; LV, left ventricle; LVEDD, left ventricular end diastolic diameter*.

Postoperative complications are presented in Figure 1B. One patient from the AAMs group was diagnosed with a sternum-wound infection postoperatively. The patient recovered from the infection with antibiotics and no further operative procedures were required. There were no sternum-wound infections in the control groups. Venous graft wound infections in legs were diagnosed from two patients in Control group I and one patient in Control group II. There were no strokes or myocardial infarctions during the study period in any of the three groups.

Discharge times from the hospital were similar between the groups. Readmission to hospital due to heart failure occurred more often in Control group I than in the AAMs group (*n* = 0, 0%; Control I *n* = 2, 33%; and Control II *n* = 1, 3%, *p* = 0.02). There were no deaths in the AAMs group during the study period of 6 months or the extended follow up time of 1 year. One patient from Control group I died of systolic heart failure during the study period (*p* = 0.046) and was lost to follow up.

Pre- and postoperative NT-pro-BNP-levels showed no statistical significance between the groups, even though IQR and median values are higher in the Control group I. Delta values demonstrated decreasing trend in AAMs group [ΔNT-pro-BNP in AAMs group −200 ng/l (range −838 to 713 ng/l) and Control group I 83 ng/l (range −160 to 424 ng/l), *p* = 0.177].

**Figure 2:**
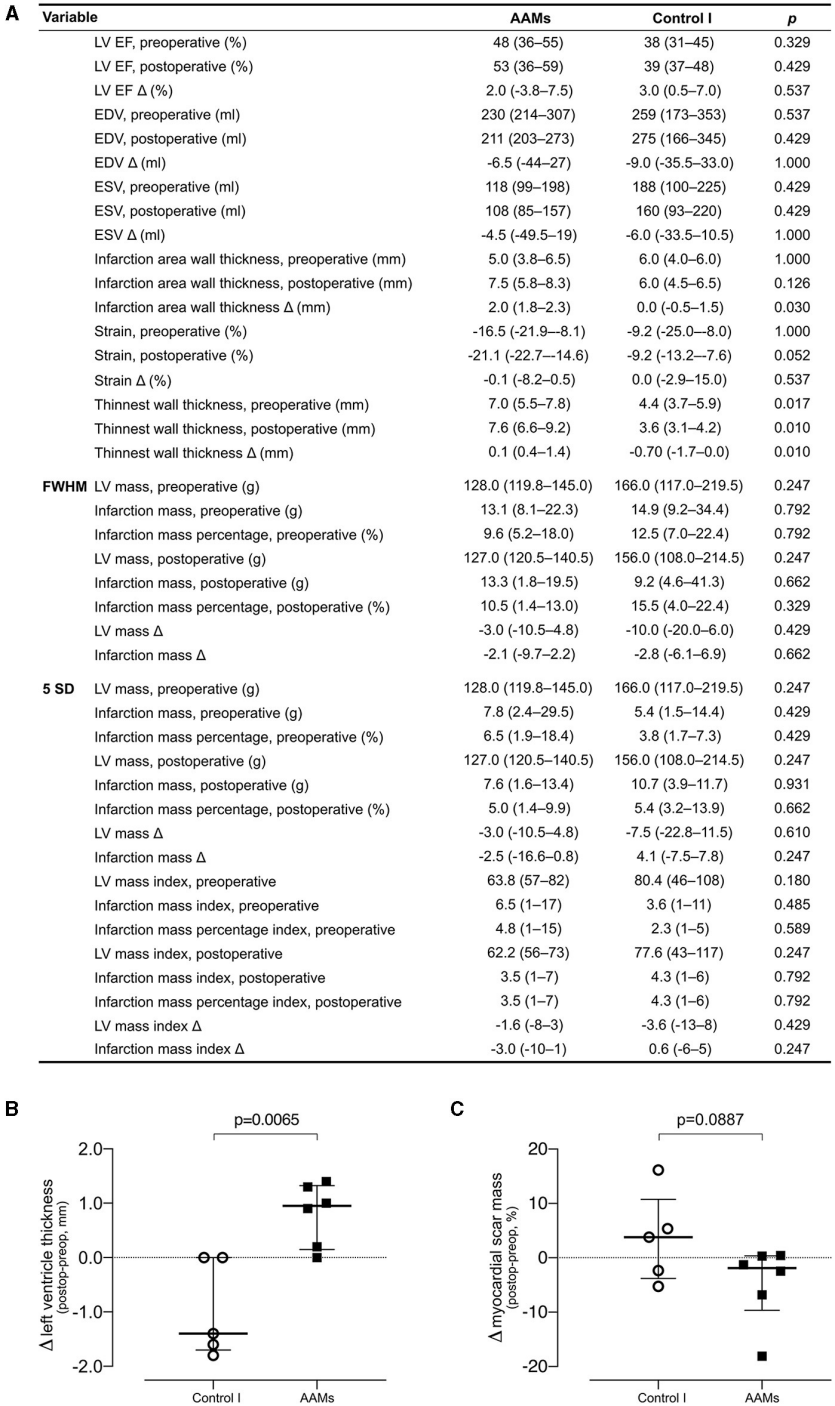
Cardiac magnetic resonance imaging—evaluation of function and structure by cardiac magnetic resonance imaging. **(A)** Comparison of the AAMs group patients with the Control group I patients. For all variables, a preoperative and post-operative value is presented, followed by the absolute change (Δ) of these values. Results presented as median and IQR, group comparison performed with the Mann Whitney *U*-test (Bonferroni adjusted *P* of 0.002). **(B)** Single parameter comparison of post-operative-preoperative change in viable left ventricle thickness at infarct scar site. **(C)** Single parameter comparison of post-operative-preoperative change in myocardial scar mass. ^1^EDV, end diastolic volume; EF, ejection fraction; ESV, end systolic volume; LV, left ventricle; FWHM, full width with half maximum; SD, standard deviation.

**Figure 3:**
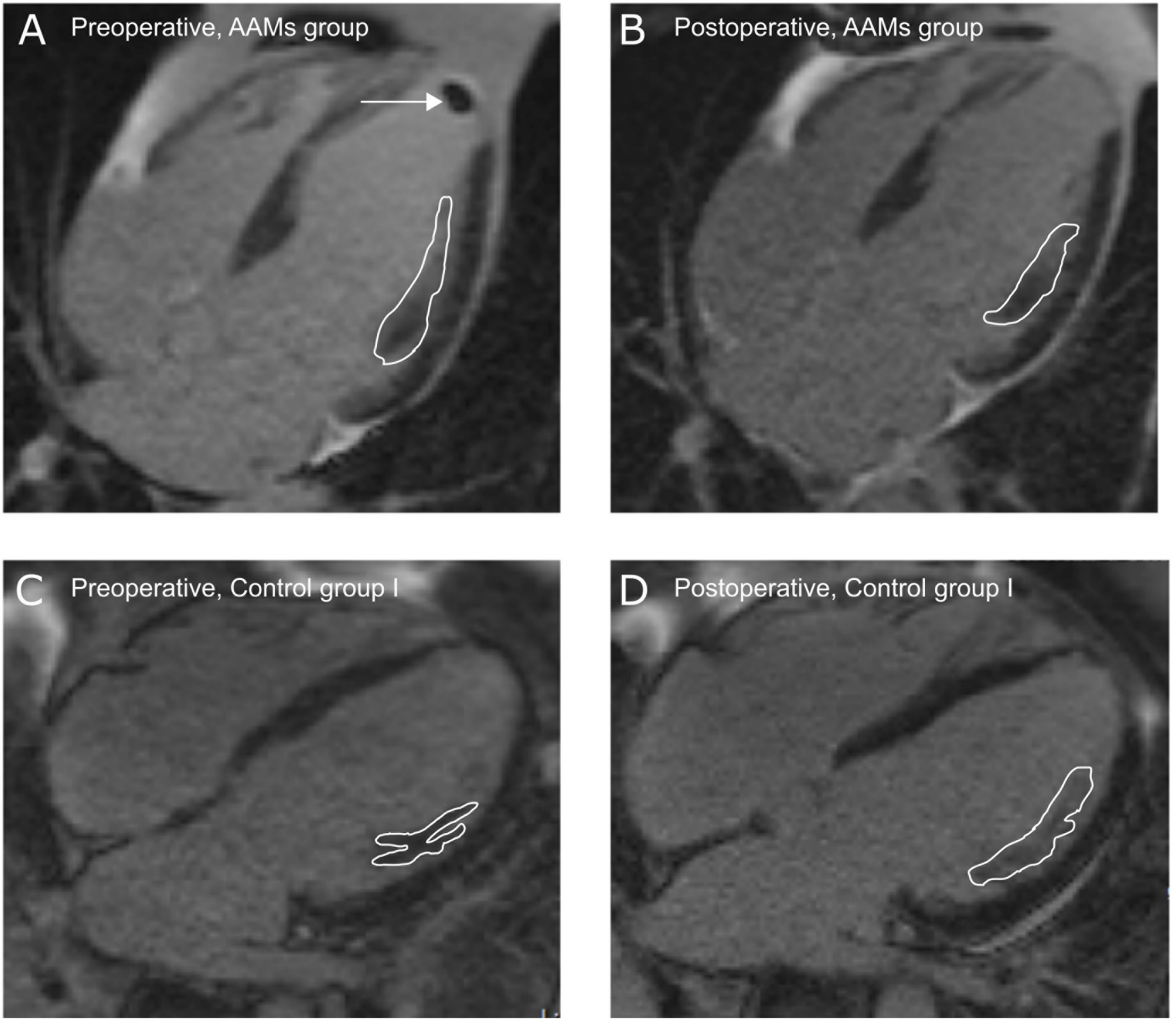
Infarct site in cardiac magnetic resonance imaging—representative pictures from cardiac magnetic resonance imaging illustrating infarct size and site (white lines) at pre- **(A,C)** and post-operative **(B,D)** time-points. **(A,B)** AAMs group and **(C,D)** Control group I. Arrow shows an apical thrombus that was removed during the coronary artery bypass surgery.

CMRI (Figure 2) was performed on patients from the AAMs group and Control group I preoperatively and at 6-months follow-up. Infarct scar was detected in all patients' preoperative CMRI analyses. Change of viable myocardial thickness at the infarcted area demonstrated a significant increase in the AAMs group [1.0 mm (range 0.2–1.3 mm)] and reduction in the Control group I [−1.4 mm (range −1.7 to 0.0 mm), *p* = 0.009]. The mainly preferred method of evaluation is the STRM of 5SD which gives a better approximation of the extent of heart infarct as compared to FWHM (16, 17). However, because many studies often use the FWHM, these results are also presented in Figure 2. Representative CMRI pictures from Control I group and AAMs group at both pre- and postoperative time points are shown in Figure 3.

**Figure 4:**
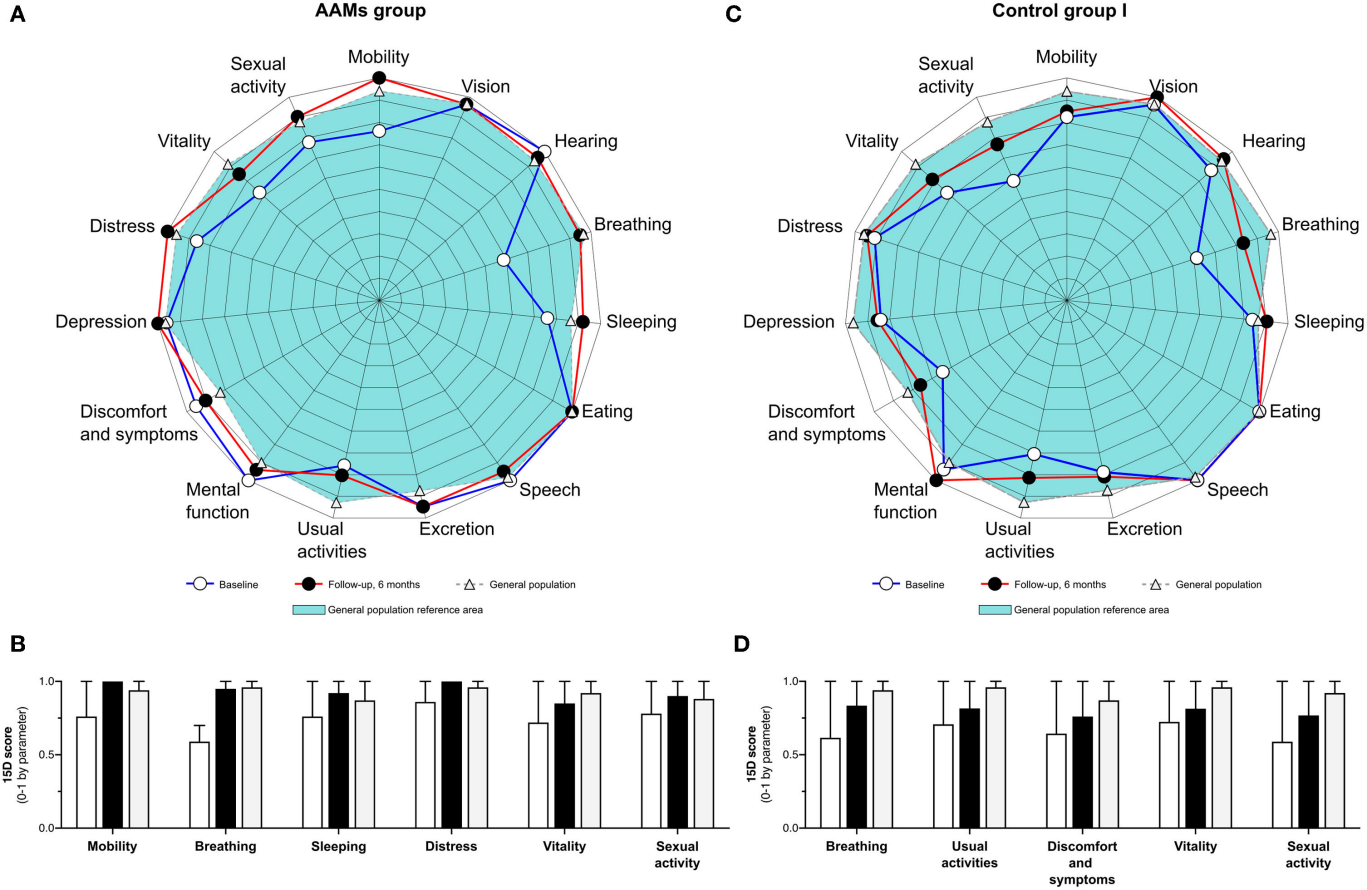
Graphical presentation of quality of life—health-related quality of life QoL in the AAMs group and the Control group I. **(A)** Graphical presentation of 15D QoL scores preoperatively and at the 6-month follow-up in the AAMs group. **(B)** Selected parameters of the 15D QoL in the AAMs group. White bars represent preoperative values, black bars represent values at 6-months follow-up and gray bars represent the respective parameter's 15D QoL score in the general population. **(C)** Graphical presentation of 15D QoL scores preoperatively and at the 6-month follow-up in the Control group I (Control I). **(D)** Selected parameters of the 15D QoL in the Control I group. White bars represent preoperative values, black bars represent values at 6-months follow-up and gray bars represent the respective parameter's 15D QoL score in the general population.

The health-related QoL of the AAMs and Control group I patients did not differ significantly at baseline or at 6 months. Similarly, neither group's QoL differed significantly from that of the reference population, that is, age-matched samples of the general population. The individual mean 15D scores for both groups are presented in Table 4. Figure 4 is a graphical presentation of all parameters at pre- and postoperative time points as compared to the general population reference. At the 6-month follow-up, the AAMs group 15D Index score mean (SD) of 0.8634 (0.07685) was slightly better than the Control group I 15D Index score mean (SD) of 0.8207 (0.15693). The mean (95% CI) difference was 0.04267 (−0.11628 to 0.20162); independent *t*-test *T*_(10)_ = 0.598, *p* = 0.006. According to the data of Sintonen (15), the Finnish age-matched adult general population (*n* = 562) has a mean (SD) 15D HRQoL index score of 0.9210 (0.07611). Compared to the reference population, the AAMs group (*n* = 6) had a mean (95% CI) difference of −0.06 (−0.27 to 0.16); independent *t*-test *T*_(5)_ = −0.69, *P* = 0.519. The Control group I's (*n* = 5) mean (95% CI) 15D index score differed −0.10 (−0.54 to 0.34); independent *t*-test *T*_(5)_ = −0.59, *p* = 0.580.

**Table 4:** Quality of life comparison—quality of life of the study and Control I group at baseline and at the 6-month follow up, with groupwise comparison.

**Quality of life D15 score**	**AAMs**	**Control I**	***p***
Mean (SD) baseline	0.863 (0.077)	0.821 (0.157)	0.568
Mean (SD) 6 month	0.938 (0.048)	0.890 (0.145)	0.463
**Difference of quality of life**	**Mean difference (95% CI)**	**T**	***p***
Study Group at baseline (*n* = 6)	−0.058 (−0.271 to 0.156)	−0.694 (df 5)	0.519
Control I Group at baseline (*n* = 6)	−0.100 (−0.536 to 0.335)	−0.592 (df 5)	0.580
Study Group at 6 months (*n* = 6)	0.0168 (−0.116 to 0.150)	0.325 (df 5)	0.758
Control I Group at 6 months (*n* = 5)	−0.031 (−0.470 to 0.409)	−0.193 (df 4)	0.856

*The mean difference comparison of both groups against the reference group of the age-matched healthy general population at baseline and at the 6-month follow-up is presented in the lower table. Results presented as mean (SD), mean difference is presented with 95% CI and groupwise comparisons were performed with the Student's t-test*.

*Abbreviations: SD, standard deviation; AAMs, autologous atrial appendage micrografts*.

## Discussion

Epicardial transplantation of AAMs was both safe and feasible to be performed during CABG surgery. The AAMs transplant did not increase the risk for any adverse effects or complications during the follow-up time. Preparing the transplant in the operation room was efficient and did not delay the operation more than the time caused by suturing it to the myocardium.

A significant increase in the change of viable myocardial thickness at the infarcted area was found in AAMs group when compared to the Control group I. This encouraging result implies that epicardial delivery of AAMs may also provide benefit as improved local functional capacity of LV. Moreover, with the AAMs transplant the risk for infarct complications such as aneurysms due to the thinning of LV are not as expected, because the infarct area is strengthened. This may also have an impact on the patients' long-term survival.

The quest for the best transplantation route for cell-therapies has been ongoing for decades. Epicardial transplantation with a matrix sheet is considered an attractive choice because of its ability to target the damaged area of myocardium, ensure better cell engraftment, and less damage for the cellular grafts than injections (18–22). The long-term survival of cells after transplantation using this method (23) is, however, in need of further studies.

Cell-based therapies are considered to exert their therapeutic effects via paracrine signaling (24, 25). The soluble factors that are secreted or released by the transplanted cells activate pathways in the host tissue that instigate intrinsic repair processes in the damaged myocardium (4, 26–28). Paracrine factors, such as cytokines, chemokines, and growth factors, drive cascades that eventually improve blood perfusion, tissue repair, and remodeling as well as inhibit hypertrophy and fibrosis (29–31). These signaling factors can also be secreted in the forms of extracellular vesicles or exosomes (32, 33). Exosomes have been proved to increase cell proliferation, reduce infarct size, increase EF (32, 33), and modify inflammation (26–28).

Paracrine activity of the AAMs may also be due to natriuretic peptides, such as atrial natriuretic peptide (ANP) and B-type natriuretic peptide (BNP), produced and secreted from the atrial myocardium (34). Secretion of natriuretic peptides is stimulated by atrial distension, stress, hypoxia, or ischemia, but may also be affected by the mechanical processing of the atrial appendage into micrografts or the epicardial microenvironment. In addition to their systemic effects regulating blood volume and vascular tone, the natriuretic peptides have been demonstrated to have paracrine anti-hypertrophic and anti-fibrotic effects on the myocardium (35). Further investigations into the paracrine factors responsible for the therapeutic effect of AAMs are warranted. Moreover, because angiogenic and cardiopoietic events are likely to occur prior to changes in myocardial viability and thus increases in scarred myocardial wall thickness, targeted investigations to evaluate the mechanisms and contribution of these events in response to AAMs treatment are in need to be addressed in further studies.

Few recent multicenter and randomized trials have shown cell-based therapies to be safe, feasible, and have long-term clinical benefit for patients. CONCERT-HF trial demonstrated a reduction in 1-year cumulative heart failure-related major adverse cardiac events after transendocardial injection of c-kit-positive cardiac cells (36). Moreover, injection of bone marrow mesenchymal cells with or without c-kit positive cardiac cells improved the patients' quality of life compared to placebo (36). In the CHART-1 trial, intramyocardially injected cardiopoietic stem cells were associated with reduced risk of death or cardiovascular hospitalization in a subpopulation of patients with advanced LV enlargement as compared to a sham procedure (37). Despite the lack of evidence in reduction of infarct scar area or improvement in LVEF, these encouraging results indicate that cell-based therapies can have long term effects in preventing progression of HF.

This study is limited by its non-randomized and open-label design as well as the low number of patients. However, for a safety and feasibility study this design can be considered appropriate. Interestingly, already in this limited patient population we identified first indications of efficacy in terms of myocardial structural changes using LGE-CMRI.

We conclude that epicardial transplantation of AAMs is safe and feasible to be performed during CABG surgery in the operating room. These results warrant further evaluation of the AAMs treatment's efficacy in larger randomized controlled trials, with changes in myocardial function and structure by LGE-CMRI as the primary efficacy endpoints. The combination of intraoperative harvesting, isolation, and transplantation of AAM cardiac micrografts shows promise as a safe, straightforward, and clinically feasible therapy during CABG surgery.

## Data Availability Statement

The original contributions presented in the study are included in the article/Supplementary Material, further inquiries can be directed to the corresponding author/s.

## Ethics Statement

The studies involving human participants were reviewed and approved by Surgical Ethics Committee of the Hospital District of Helsinki and Uusimaa (number 180/13/03/02/13). The patients/participants provided their written informed consent to participate in this study.

## Author Contributions

AN, TN, ML, TP, RS, EK, AH, and AV conceived the study design and coordination and helped to draft the manuscript. ML and EK designed and developed the cell graft. KT and TJ operated the study group patients. JS performed the analysis and helped to draft the manuscript. HS provided the QoL reference data and helped with the analysis. AN and SM drafted the manuscript. All authors contributed to the interpretation of the data, read, and approved the final manuscript.

## Funding

This work was supported by AH, MK, AV, and AN by Finnish Government Block Grants for Clinical Research (Grant Nos. TYH Y2016SK013, TYH2016211, TYH2015311, TYH2019266, and Y1016SK017), EK by Finnish Funding Agency for Technology and Innovation (Grant No. 40033/14), and AN by Finnish Society of Angiology.

## Conflict of Interest

AN and EK are stakeholders in EpiHeart Ltd. developing medical devices for the operating room. HS is the developer of the 15D. The remaining authors declare that the research was conducted in the absence of any commercial or financial relationships that could be construed as a potential conflict of interest.

## Publisher's Note

All claims expressed in this article are solely those of the authors and do not necessarily represent those of their affiliated organizations, or those of the publisher, the editors and the reviewers. Any product that may be evaluated in this article, or claim that may be made by its manufacturer, is not guaranteed or endorsed by the publisher.
