# Supplementary material for: Epicardial Transplantation of Autologous Cardiac Micrografts During Coronary Artery Bypass Surgery
Source: Front Cardiovasc Med. 2021 Sep 14;8:726889. doi: 10.3389/fcvm.2021.726889 (PMC8476794; doi:10.3389/fcvm.2021.726889)
Supplement: Supplementary file 1 [file Data_Sheet_1.DOCX]

***Supplementary material***

**1 List of abbreviation**:

AB-BE = Arterial blood base excess

AAMs = Autologous atrial appendage micrografts

ANP = Atrial natriuretic peptide

ARB = Angiotensin receptor blocker

BMI = Body Mass Index

CABG = Coronary artery bypass grafting

CCB = Calcium channel blockers

CMR = Cardiac magnetic resonance imaging

COPD = Chronic Obstructive Pulmonary Disease

CVP = Central venous pressure

ECG = Electrocardiogram

ECM = Extracellular matrix

EDV = End diastolic volume

ESV = End systolic volume

EF = Ejection fraction

FWHM = Full-width at half-maximum

GFR = Glomerular filtration rate

Hb = Hemoglobin

LA = Left atrium

LBBB = Left bundle branch block

LV = Left ventricle

LVEDD = Left ventricular end diastolic diameter

MAP = Mean arterial pressure

MRA = Mineralocorticoid receptor antagonists

NYHA = New York Heart Association

NT-PRO-BNP = N-terminal pro-B-type natriuretic peptide

PCI = Percutaneous coronary intervention.

SD = Standard deviation above the mean

SI = signal intensity

SPO2 = Peripheral capillary oxygen saturation

STRM = signal threshold versus reference mean

**2 AADC Consortium****

Annu Nummi; Juhani A Stewart; Tommi Pätilä; Tuomo Nieminen; Milla Lampinen; Severi Mulari; Miia L Lehtinen: Sari Kivistö; Erika Wilkman; Kari Teittinen; Mika Laine; Juha Sinisalo; Markku Kupari; Esko Kankuri; Tatu Juvonen; Antti Vento; Raili Suojaranta; Ari Harjula; Eero Mervaala; Matti Kankainen; Jari Laurikka; Shengshou Hu; Zhe Zheng; Xie Yanbo
